# A Relationship between NTP and Cell Extract Concentration for Cell-Free Protein Expression

**DOI:** 10.3390/life11030237

**Published:** 2021-03-13

**Authors:** Katsuki Takahashi, Gaku Sato, Nobuhide Doi, Kei Fujiwara

**Affiliations:** Department of Biosciences & Informatics, Keio University, 3-14-1 Hiyoshi, Kohoku-ku, Yokohama 223-8522, Japan; katsuki@z6.keio.jp (K.T.); sato-gaga.1212@keio.jp (G.S.); doi@bio.keio.ac.jp (N.D.)

**Keywords:** cell-free protein synthesis (CFPS), NTP requirement, reconstitution of living cells, cell extract, synthetic biology

## Abstract

The cell-free protein synthesis (CFPS) that synthesizes mRNA and protein from a template DNA has been featured as an important tool to emulate living systems in vitro. However, an obstacle to emulate living cells by CFPS is the loss of activity in the case of usage of high concentration cell extracts. In this study, we found that a high concentration of NTP which inhibits in the case of lower concentration cell extract restored the loss of CFPS activity using high concentration cell extracts. The NTP restoration was independent of the energy regeneration system used, and NTP derivatives also restored the levels of CFPS using a high concentration cell extract. Experiments using dialysis mode of CFPS showed that continuous exchange of small molecule reduced levels of NTP requirement and improved reaction speed of CFPS using the high concentration of cell extract. These findings contribute to the development of a method to understand the condition of living cells by in vitro emulation, and are expected to lead to the achievement of the reconstitution of living cells from biomolecule mixtures.

## 1. Introduction

Cell-free protein synthesis (CFPS) that synthesizes proteins from template DNA through transcription-translation machinery extracted from cells has been widely used in applications and analysis of biological systems in living cells [1,2,3]. Because of its easiness of handling and ability to express wide varieties of proteins, CFPS is widely applied to protein engineering including directed evolution of proteins and synthesis of cytotoxic proteins [2,3,4,5,6,7]. Since CFPS has the ability to reconstitute biochemical systems from DNA mixtures, it has been also featured as an important material for research toward rebuilding living cells in vitro [8,9,10,11,12,13]. As another application, CFPS has been utilized for protein folding studies [14,15], membrane insertion analysis [16,17], and elucidation of molecular machinery in living cells such as translational arrest [18]. The range of proteins that CFPS can express is wide, and it has been shown that CFPS can synthesize more than half of *Escherichia coli* [19] and *Homo Sapiens* proteins [20].

In spite of the versatile usage of CFPS, its protein expression rate is much slower than that in living cells. For example, *E. coli* cells that contain more than 100 mg/mL of protein [21,22] self-replicates within 30 min, suggesting that the cell synthesizes at least 100 mg/mL of protein per 30 min. Because the maximum CFPS speed is less than 1 mg/mL proteins per hour so far [2,23], which is considered to be 1/100 of that in the cell. One of the reasons for this slow protein synthesis is that the rate of enzymatic reaction depends on the amount of enzyme. For typical CFPS, cell extracts of 10–20 mg/mL are used [23,24,25,26,27,28], which is about 1/10 of the intracellular concentration. This fact suggests that the protein synthesis rate can speed up by using high concentration cell extract (HCCE). However, it has been shown that the efficiency of CFPS is rather reduced when using HCCE [29,30].

In this study, we pursued the conditions for efficient CFPS using HCCE. As a result, it was shown that the amount of high-concentration NTP, which inhibits in the case of lower concentration cell extracts [31,32], conversely improves the reaction efficiency in HCCE. In addition, from analysis using an NTP element (ATP and its derivatives) and CFPS under dialysis condition, this NTP amount dependence is derived from physicochemical reaction environments and metabolism rather than energy sources. These results indicate that NTP concentration is an important factor for efficient CFPS using HCCE. It is expected that the results of this study will lead to developing an enhanced cell-free system that transcribes and translates at the same efficiency and rate as living cells, and will contribute to the realization of the reconstitution of living cells in vitro.

## 2. Materials & Methods

### 2.1. Chemicals

Chemicals used in this study were mainly purchased from Nacalai tesque (Kyoto, Japan). Lysine, 3-phospho-D-glyceric acid barium salt dihydrate, folinic acid, and CTP were purchased from Tokyo Chemical Industry (Tokyo, Japan). Potassium glutamate, adenosine, and cysteine were purchased from Sigma-Aldrich (St. Louis, MO, USA). Creatine kinase was purchased from Roche (Basel, Switzerland). ATP, GTP, NAD^+^, and creatine phosphate were purchased from FUJIFILM Wako Pure Chemical (Osaka, Japan). Tryptone and Yeast Extract were purchased from BD (Franklin Lakes, NJ, USA). UTP was purchased from Affymetrix (Santa Clara, CA, USA).

### 2.2. Preparation of Cell Extract

The protocol of cell extract preparation from *Escherichia coli* BL21-codonPlus(DE3)-RIPL (Agilent Technologies, Santa Clara, CA, USA) was according to the previous study [28]. Cells were cultivated in 2 × YTPG medium [16 g/L Tryptone, 10 g/L Yeast Extract, 5 g/L NaCl, 40 mM Na_2_HPO_4_, 22 mM NaH_2_PO_4_, 100 mM Glucose] and harvested by centrifugation (15,000× *g* for 3 min at 4 °C) at OD_600_ = 3.0. The pellet was suspended in 400 mM sucrose and added 400 μg/mL lysozyme chloride at the final. After incubation on ice for 1 h, the cells were washed with 400 mM sucrose twice and were suspended in 1.5 times the volume of S30A buffer [10 mM Bis-Tris (pH 7.0), 60 mM potassium glutamate, 10 mM magnesium acetate]. The cells were frozen by liquid nitrogen for 15 min and thawed in ice water. After centrifugation (30,000× *g* for 60 min at 4 °C), the supernatant was incubated for 90 min at 37 °C and centrifugated 10,000× *g* for 15 min at 37 °C. The supernatant was concentrated 5 times using Amicon Ultra-15 Centrifugal Filter Units 10 kDa (Merck Millipore, Billerica, MA, USA). The extract was transferred to Spectra/Por^®^ molecular porous membrane tubing MWCO: 6–8 kDa (Spectrum Laboratories Inc., Rancho Dominguez, CA, USA), and dialyzed overnight against 100 times volume of S30B buffer [10 mM Bis-Tris (pH 7.0), 60 mM potassium glutamate, 14 mM magnesium acetate] at 4 °C. The tube was transferred to the same volume S30B buffer and was further dialyzed at 4 °C for 4 h. The extract was transferred to a 50 mL tube and centrifugated 10,000× *g* for 15 min at 4 °C. The supernatant was concentrated by using the Amicon filter (10 kDa), and then dispensed to 1.7 mL tubes. The tubes were frozen in liquid nitrogen and stocked at −80 °C. The protein concentration of cell extract was determined by Pierce^®^ BCA Protein Assay Kit (Thermo Fisher Scientific, Waltham, MA, USA). Highly concentrated cell extract (>120 mg/mL) was stored, and it was diluted with the S30B buffer to the concentration mentioned prior to use. We should note that cell extract concentrations described in this study indicated protein concentrations in the cell extracts, which differs from the description in our previous study which uses macromolecule concentration (RNA + proteins) as the cell extract concentration [29].

### 2.3. Preparation of NTP Mixture

As the NTP mixture in this study, adenosine 5′-triphosphate disodium salt (ATP), guanosine 5′-triphosphate disodium salt (GTP), cytidine 5′-triphosphate disodium salt (CTP), and Uridine 5′-triphosphate trisodium salt (UTP) were dissolved with ultrapure water and mixed together (150 mM ATP, 150 mM GTP, 100 mM CTP, 100 mM UTP). These were stocked as NTP 150× in −30 °C. In this study, 1.0 mM ATP, 1.0 mM GTP, 0.67 mM CTP, 0.67 mM UTP was set as NTP 1×.

### 2.4. Cell-Free Protein Synthesis Reaction

Unless otherwise stated, cell free protein synthesis (CFPS) reaction was carried out as follows. The reaction contained 20–80 mg/mL cell extract, NTPs as indicated, 1 mM each amino acids, 67.8 µM folinic acid, 1 mM cAMP, 1 mM NAD^+^, 0.5 mM coenzyme A (CoA), 2 mM spermidine, 120 mM potassium glutamate, 100 mM Bis-Tris (pH 7.0), 14 mM magnesium acetate, 10 nM sfGFP plasmid [28], and energy source [50 mM glucose, 50 mM 3-Phosphoglyceric acid (PGA), 50 mM phosphoenolpyruvic acid (PEP), or 80 mM creatine phosphate. 60 mM K_2_HPO_4_ and 400 μg/mL creatine kinase were additionally supplied in the cases of glucose and creatine phosphate, respectively]. The template plasmid was purified by using Mini Plus™ Column (Viogen, New Taipei City, Taiwan) and a home-made miniprep solution of QIAprep Spin Miniprep Kit (Qiagen, Dusseldorf, Germany). CFPS was performed by incubating the reaction mixture (5 μL) in 0.2 mL tubes at 30 °C for 6 h if not mentioned. Dialysis mode reactions were performed using Slide-A-Lyzer^®^ MINI Dailysis Units 10,000 MWCO (Thermo Fisher Scientific, Waltham, MA, USA). The volume of reaction mixture (the same as the mixture in batch reaction except volume) was 50 μL, and the feeder solution which is the same as the reaction mixture except cell extracts and template DNA were omitted was 1.6 mL. CFPS was performed by incubating the reaction mixture at 30 °C and the feeder solution was stirred by a magnetic stirrer. 

The levels of sfGFP synthesis were evaluated by fluorescent intensities of sfGFP band using non-boiling SDS-PAGE [28]. The reaction solution was mixed with the same volume 2× sample buffer [150 mM Tris-HCl (pH 7.6), 4% SDS, 10% sucrose, 0.01% BPB], and applied to SDS-polyacrylamide gel. After electrophoresis, fluorescent bands of the gel were captured by ChemiDoc Touch MP (Bio-Rad Laboratories, Hercules, CA, USA). The amount of expressed sfGFP was estimated from standard curves obtained by purified sfGFP from the same gel using imageJ. 

### 2.5. Simulation Using PURE Simulator

PURE system simulator constructed by another group [33] was used as the simulator of a protein synthesis reaction in this study. PURE simulator written in SBML (Systems Biology Markup Language) format was downloaded from the website of the developer (https://sites.google.com/view/puresimulator, accessed on 11 March 2021) and was executed in MATLAB (Mathworks, Natick, MA, USA). All simulations were performed for 1000-s reaction using ODE15s. The concentration of elements in PURE system was varied as described in the main text.

## 3. Results

**Figure 1:**
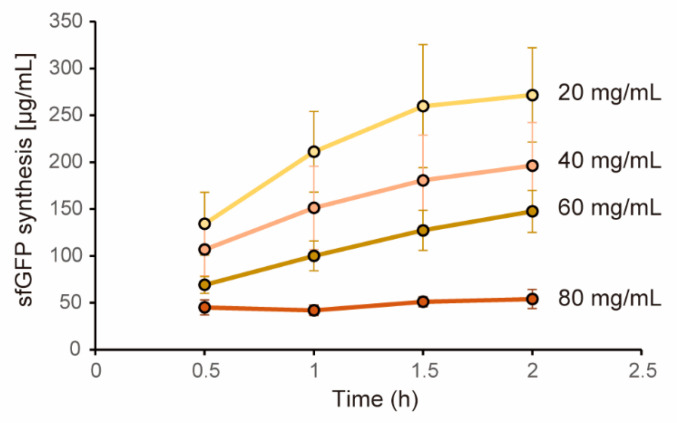
Dependence of CFPS levels on cell extract concentration. Expression levels of sfGFP were quantified periodically (0.5 h, 1 h, 1.5 h, 2 h) from fluorescence levels of sfGFP bands obtained by non-boiling SDS-PAGE. Concentration of sfGFP was determined from calibration curve obtained by purified sfGFP. CP-CK system was used as an energy regeneration system. Volumes of reaction mixtures are the same irrespective of the cell extract concentrations. Error bars indicate standard error of 3 times experiments performed in different days.

In this study, we utilized cell extract as a protein expression machinery. The cell extract we used was prepared by LoFT method which disrupts cells by biochemical treatments [28]. First, we tested whether the cell extract by LoFT method shows similar dysfunction at higher concentration to the case in the cell extract prepared by a sonication method [29]. CFPS levels under various cell extract concentrations were quantified by using plasmid encoding sfGFP under a strong σ_70_ promoter as the template DNA. In this experiment, we used creatine phosphate-creatine kinase system (CP-CK) as the energy source as similar to our previous study [29]. Expression levels of sfGFP was quantified by using non-boiling SDS-PAGE to exclude autofluorescence which is extremely problematic in the case of the high concentration cell extract (HCCE). Consequently, time plots of sfGFP expression clearly showed that higher concentration of cell extract showed lower expression levels of sfGFP (Figure 1). In the case of 80 mg/mL cell extract, expression levels of sfGFP decreased by 80%. The lowering levels of sfGFP expression was in proportion to the cell extract concentration used. From these results, we defined cell extract with 80mg/mL or more as the HCCE in this study. 

Next, we investigated the cause of dysfunction of CFPS activity found in HCCE. To explain the lower CFPS activity found in HCCE, we tested two possibilities. The first possibility we tested was that inadequate small molecule environments such as magnesium concentration reduced CFPS activity. A previous study showed a high concentration of magnesium can restore the decrease of CFPS activity using HCCE [34]. To test the possibility, CFPS activity of HCCE under various magnesium concentrations was investigated. However, although the increase of magnesium concentration improved CFPS activity of 30 mg/mL cell extract, the improvement was not found in the case of 40 and 50 mg/mL cell extract (Supplementary Figure S1). 

**Figure 2:**
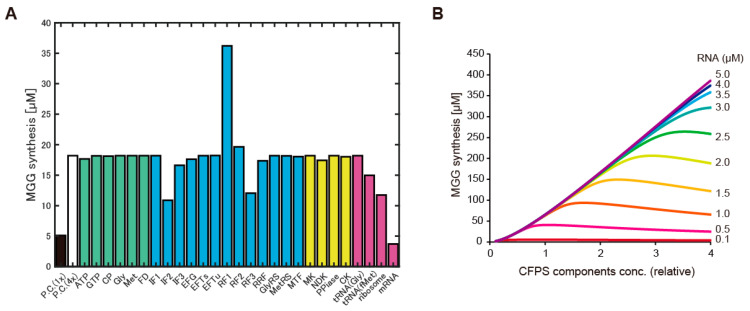
Analysis of shortage elements by using a biochemical simulator of CFPS. (**A**): Protein expression levels after 1000 s simulation using the PURE system simulator. P.C. (1×) indicates the developer condition of the PURE simulator. P.C. (4×) indicates concentration of all elements was 4 times higher than the developer condition. Others indicate the molecule that was omitted from the 4 times increase of the concentration. The y-axis indicates levels of peptide synthesis and MGG indicates the peptide Met-GlyGly which is the final product of the PURE system simulator. Colors of bars indicate group of the molecules (green: small molecules, blue: translation elements, yellow: other proteins, magenta: RNAs). (**B**): Relation among concentration of CFPS elements, protein synthesis levels after 1000 s simulation, and RNA concentration.

The second possibility we tested was dysfunction or shortage of some elements of CFPS using HCCE. Because near 1000 reactions are associated with CFPS, we firstly surveyed the cause of dysfunction by using biochemical simulation. A previous study has developed a detailed biochemical simulation model, PURE system simulator, in which all translational reactions (~968) were implemented [33]. First, we supposed that the low CFPS activity in the case of HCCE was derived from dysfunction of some elements, and therefore, the effect of dysfunction was emulated by the simulator. To emulate the dysfunction, concentrations of all elements except one was increased four-fold, and the effect of the concentration change of small molecules or macromolecules on the CFPS activity in the simulator were widely surveyed one by one. The dysfunction emulation revealed reduction of several molecules negatively affected CFPS activity. The most critical element was RNA as the template (Figure 2A). Relatively lower setting of RNA levels canceled 4-fold increase of other elements. This point was further analyzed by varying RNA concentration with the variation of CFPS concentrations. Consequently, the simulation suggested that more CFPS elements required more RNA levels for efficient CFPS activity (Figure 2B).

**Figure 3:**
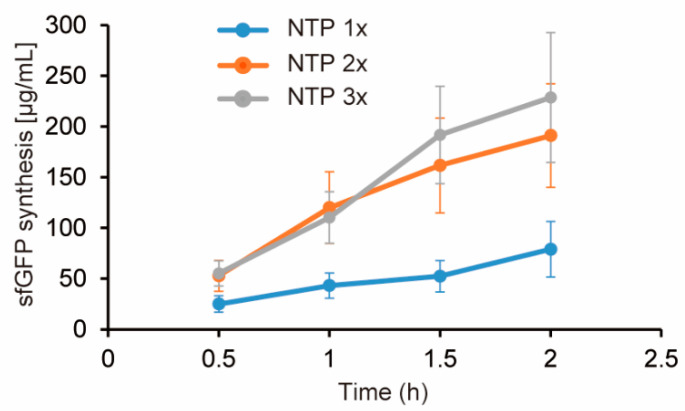
Increase of NTP concentration restores CFPS activity in high concentration cell extract. NTP 1× indicates normal NTP concentration described in Methods. NTP 2× and 3× indicate concentration of all NTP elements was doubled and tripled compared with the normal NTP concentration. Cell extract concentration used was 80 mg/mL. Error bars indicate standard error of 4 times experiments performed in different days.

Because the PURE simulator lacks a model of transcription, only RNA concentration is the variable. On the other hand, CFPS that transcripts RNA from DNA, and the level of RNA is able to be tuned by three factors: DNA, RNA polymerase, and the substrate (NTP). Because DNA is excessively added and RNA polymerase levels is proportion to the concentration of cell extracts, we focused on NTP levels to increase RNA levels. Actually, a previous study showed higher NTP concentration increases RNA levels (32). Concentration of all NTP elements (ATP, GTP, UTP, CTP) was doubled, and CFPS activity were compared with the case of normal concentration of NTP. In contrast to the inhibitory effect of higher concentration of NTP found in the previous study using lower concentration of cell extracts, doubled NTP concentration restored CFPS activity in the case of HCCE, and the expression levels sfGFP was tripled (Figure 3). Tripled NTP concentration provided similar results with the doubled NTP concentration experiment (Figure 3).

**Figure 4:**
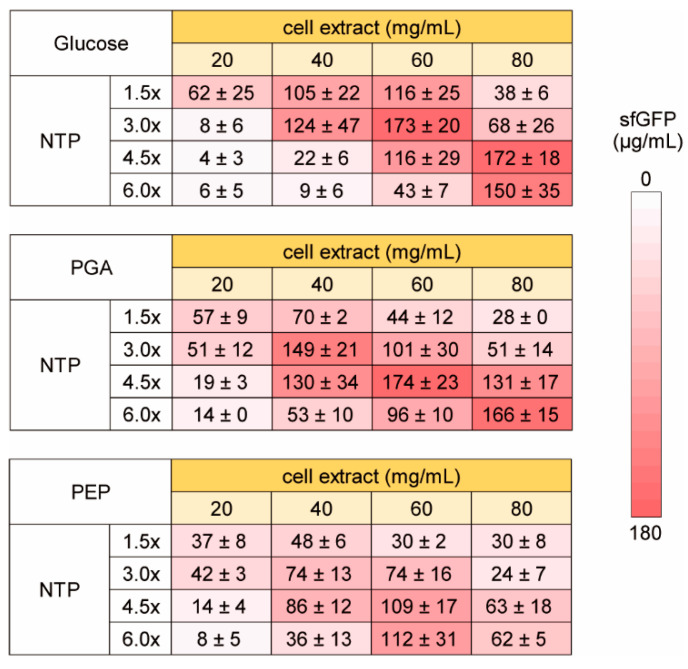
Energy and NTP dependence of high concentration cell extract on CFPS activity. The levels of sfGFP synthesis (µg/mL) on averages and the standard errors of 3 times experiments performed on different days are shown. Raw data are shown in Supplementary Figure S2.

Next, we investigated the dependence of the NTP requirement of HCCE for efficient CFPS activity on energy sources. For CFPS reaction using cell extract, entire or partial glycolysis pathway have been widely used for energy regeneration in addition to the CP-CK system used above [24,25,26,35,36]. Therefore, we tested three energy regeneration systems using glycolysis. One is glucose, which requires phosphate and NAD^+^ for energy synthesis and uses whole pathway of glycolysis. The others are PGA and PEP, which use downstream pathway of glycolysis. In this experiment, we used from 1.5 to 6 times higher concentration of NTP to investigate NTP dependence clearly (Figure 4). In all glycolysis substrates tested, increase of NTP concentration inhibited CFPS activity under lower cell extract conditions. Similarly, increase of cell extract concentration inhibited CFPS activity at lower NTP concentrations. In contrast, higher cell extract CFPS requires higher concentration of NTP for efficient sfGFP synthesis as similar to the case of the CP-CK usage. Interestingly, although the total trend of NTP and cell extract dependence was similar among glucose, PGA, and PEP, optimum concentrations were varied. Therefore, it was suggested that some metabolism other than RNA synthesis were associated with NTP dependence of HCCE on CFPS activity.

We can raise several possibilities of the reason of the requirement of NTP. One is mRNA levels, which we originally supposed. The others are simple energy requirement, hydrotrope effect of ATP [37], and other unconsidered environmental effects for HCCE. To check these points, we tested the effect of ATP and its derivatives on the NTP requirement. If mRNA is the major cause, excess ATP supplementation does not restore because GTP, CTP, and UTP are also necessary for RNA synthesis. If energy requirement is the cause, ATP works like NTP but ADP does not. If hydrotrope effect is the cause, dATP also works as ATP. To consider other unconsidered environmental effects for HCCE, adenosine, which is similar to ATP but does not have phosphate was tested. 

**Figure 5:**
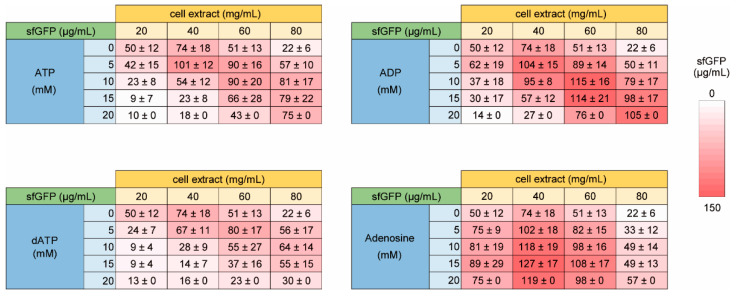
Effect of ATP and its derivative of high concentration cell extract on CFPS activity. The levels of sfGFP synthesis (µg/mL) on averages and the standard errors of 3 times experiments performed on different days are shown. Raw data are shown in Supplementary Figure S3.

Consequently, ATP and ADP showed similar trend to the case of NTP (Figure 5). The results suggested that RNA and initial energy source was not the cause of the requirement of NTP. Compared with ATP, a higher concentration of ADP was required for the restoration of CFPS activity using HCCE. Since ADP does not work initial energy but works a substrate in energy regeneration of glycolysis, the results do not deny the possibility of that restoration of CFPS activity using HCCE by ATP and ADP is based on energy supplementation during CFPS. Because a previous study reported that ADP shows weaker hydrotrope effect at the same concentration [37], we considered that the hydrotrope effect was the reason why HCCE requires a higher concentration of ADP. To check the point, we tested the effect of dATP, which is not involved in the energy regeneration process of glycolysis but should cause the hydrotrope effect. In the case of dATP, higher concentration dose actually suppressed the NTP requirement as similar to ATP or ADP (Figure 5), and its trend was rather similar to the case of ATP. However, the restoration levels of CFPS activity by dATP were relatively lower than NTP, ATP, and ADP. Although interpretation of the result was not clear, it was suggested that both hydrotrope effect and enhanced energy regeneration were involved in the NTP requirement of HCCE. On the other hand, the addition of adenosine increased sfGFP expression all concentration of cell extract tested (Figure 5). Although the reason why adenosine improved CFPS activity was unclear, the result suggested that other unconsidered environmental effects are also associated with NTP requirement of HCCE.

**Figure 6:**
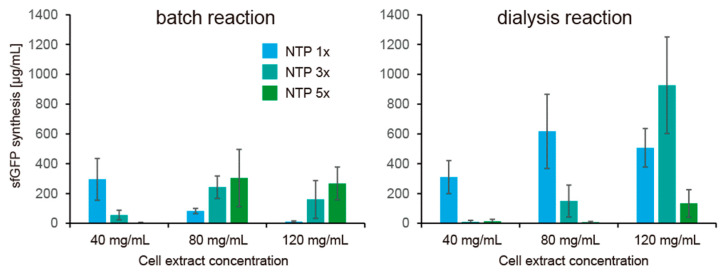
NTP dependence of CFPS using highly concentrated cell extract under dialysis mode. NTP 1× indicates normal condition described in Methods, and NTP 3× and 5× indicate concentration of all NTP elements (ATP, GTP, CTP, UTP) are 3 times and 5 times higher than the normal concentration. Error bars indicate the standard errors of 3 times experiments performed on different days. Levels of sfGFP synthesis after 2 h reaction are shown. Raw data are shown in Supplementary Figure S4.

Finally, we tested the effect of continuous feeding of small molecules and removal of unnecessary molecules produces during CFPS. For the purpose, small molecules were continuously exchanged with a fresh CFPS solution by dialysis. In the dialysis mode, it was expected that concentrations of small molecules were kept constant. We investigated whether the NTP requirement of HCCE for CFPS activity remains or diminishes under the dialysis condition. In this reaction, glucose was used as an energy regeneration system, and sfGFP expression levels were quantified after 2 h reaction. In contrast to the batch mode, the dialysis mode showed weaker dependence of NTP concentration on CFPS activity using HCCE (Figure 6). In contrast to that batch mode CFPS showed higher cell extract concentration required higher NTP concentration for sfGFP expression, dialysis mode of CFPS using 80 mg/mL cell extract showed the highest sfGFP expression at 1× NTP (normal NTP concentration) among conditions tested. In the case of dialysis mode of CFPS using 120 mg/mL cell extract, 3× NTP concentration showed better sfGFP expression than the cases of 1× or 5× NTP. These results implied that NTP consumption was strong in the batch reactions, and therefore, HCCE requires higher concentration of NTP. 

Another remarkable finding was a practical improvement of CFPS activity by the dialysis reaction. A problem of the CFPS using HCCE was little practical improvement of CFPS activity compared with the CFPS using a lower concentration of cell extract irrespective of the restoration by excess NTP supplementation. Especially, its slower protein synthesis compared with the speed expected by enzymology was a major problem. On this point, the dialysis mode of CFPS showed that a higher concentration of cell extract synthesizes a higher amount of sfGFP in shorter time reactions, as expected by enzymology. We should note that, although the error bars in Figure 6 was large, the trend was kept among experiments on different days, and we have never observed that protein synthesis reaches 1 mg/mL in 2 h in the case of the batch-mode reaction performed many times in this study. These pieces of evidence indicate that our conclusion is based on statistical significance to some extent. These results indicated that low CFPS activity in the case of HCCE was due to the metabolism of the substrates and improvement of metabolism is important for increasing the protein expression using HCCE.

## 4. Discussion

Previous researches have shown that fine-tuning of concentration of small molecules is necessary for the improvement of cell-free protein synthesis using cell extract [36]. For this improvement, much attention was paid to energy regeneration systems. Especially, usage of glycolysis and its phosphate donor was a key to improving the CFPS activity [24,25,26,35,36]. Less weight attention has been paid to other small molecules for CFPS, but several studies have shown that the concentration of some molecules is critical for efficient CFPS reactions [36,38]. For example, higher NTP concentration inhibits CFPS activity, and Mg concentration modulates the range of NTP concentration for inhibition [32]. However, the reason why the increase of cell extract concentration lowered CFPS activity was still elusive, and disturbance of metabolism by elements in cell extract was considered to be associated with the lower CFPS activity. On this point, our present study clearly showed that a higher concentration of NTP, which inhibits CFPS activity using a lower concentration of cell extract, is rather necessary for efficient CFPS activity using highly concentrated cell extract in batch mode.

Our results indicate that the restoration of protein synthesis levels of CFPS using HCCE by excess NTP supplementation is derived from complex effects. Replacement of NTP with ATP and its derivatives suggested that both gaining energy regeneration levels and tuning physicochemical environment by NTP such as hydrotrope effect are involved in the restoration. Shifts of optimum NTP concentration and increase of protein synthesis speed found in the dialysis mode experiments indicated that keeping NTP levels higher than a threshold and removal of secondary produced molecules such as pyrophosphate are needed for efficient CFPS using HCCE. Since cell extracts are expected to use NTP in many metabolisms other than energy regeneration, excess NTP consumption is another reason for the NTP requirement of HCCE. As another possibility, it should be considered that the low CFPS activity of HCCE is associated with high viscosity and/or macromolecular crowding effects that modulate collision rates of molecules. On this point, our results suggested that these physicochemical effects are not problematic at least under dialysis conditions. However, NTP consumption and hydrotrope effects can compensate for these physicochemical effects in batch-mode. These points should be clarified in future studies.

As our recent study showed that CFPS has the ability to reconstitute transcriptome and proteome to some extent [10], the improvement of HCCE activity was an urgent task toward the reconstitution of living cells. However, lower activity of CFPS using HCCE was a remarkable difference between in vitro and in vivo. Therefore, the most striking result in this study is the finding of the restoration of CFPS activity using HCCE by increasing the concentration of NTP. Our result suggests that dialysis mode with relatively higher NTP concentration is the key for efficient CFPS activity of HCCE. Dialysis needs a semipermeable membrane, and it has been shown that liposome with membrane proteins works as a semipermeable membrane to enable continuous CFPS reaction as the dialysis mode in vitro [39]. In the liposomes harboring membrane proteins for small molecules exchange and encapsulating adequate concentration of NTP, HCCE, and genome, CFPS is expected to synthesize RNA and proteins as the levels of living cells do. If this level of liposome were once developed, it would be possible to analyze the gaps between mixtures of biomolecules as a material to mimic living cells and living cells themselves. We believe that this kind of analysis will pave the way to elucidate an open question, “what is life”.

## Data Availability

Not applicable.

## Supplementary Materials

The following are available online at https://www.mdpi.com/2075-1729/11/3/237/s1, Figure S1: Mg dependence of high concentration cell extract on CFPS activity.Averages and standard error of 3 times experiments performed in different days were shown; Figure S2: Energy source dependence of high concentration cell extract on CFPS activity. Raw data of Figure 4 are shown. The values indicate the levels of sfGFP synthesis (μg/mL); Figure S3: Effect of ATP and its derivative of high concentration cell extract on CFPS activity. Raw data of Figure 5 are shown. Setting of the heat map is the same as in Figure 5. The values indicate the levels of sfGFP synthesis (μg/mL); Figure S4. NTP requirement of high concentration cell extract for cell-free protein synthesis. Raw data of dialysis-mode results in Figure 6 are shown.
