# Supplementary material for: A Relationship between NTP and Cell Extract Concentration for Cell-Free Protein Expression
Source: Life (Basel). 2021 Mar 13;11(3):237. doi: 10.3390/life11030237 (PMC7999496; doi:10.3390/life11030237)
Supplement: Supplementary file 1 [file life-11-00237-s001.pdf]

Supplementary Information

A relationship between NTP and cell extract concentration for cell-free protein expression

Katsuki Takahashi, Gaku Sato, Nobuhide Doi, Kei Fujiwara*

Supplementary Figures S1-S4

GFP synthesis (µg/mL)		cell extract (mg/mL)			
		20	30	40	50
Mg conc (mM)	5	20±3	102±15	144±9	154±17
	10	205±37	298±27	149±11	118±6
	15	325±38	329±44	221±18	142±31
	20	198±26	332±39	191±9	103±43
	25	71±35	328±41	93±8	28±17

Supplementary Figure S1. Mg dependence of high concentration cell extract on CFPS activity.
Averages and standard error of 3 times experiments performed in different days were shown.

Day 1

Glucose		cell extract (mg/mL)			
		20	40	60	80
NTP	1.5x	48.3	147.8	155.2	32.8
	3.0x	3.5	124.1	212.9	110.5
	4.5x	1.7	21.0	73.6	172.1
	6.0x	1.9	6.3	40.8	183.1

PGA		cell extract (mg/mL)			
		20	40	60	80
NTP	1.5x	62.4	66.7	20.8	27.2
	3.0x	71.5	147.3	95.7	78.6
	4.5x	24.3	183.4	156.7	118.5
	6.0x	14.9	52.7	96.5	140.4

PEP		cell extract (mg/mL)			
		20	40	60	80
NTP	1.5x	37.5	47.2	26.8	27.1
	3.0x	47.2	86.1	85.9	35.1
	4.5x	21.9	72.6	101.6	58.4
	6.0x	18.0	59.2	124.4	54.9

Day 2

Glucose		cell extract (mg/mL)			
		20	40	60	80
NTP	1.5x	111.2	92.5	69.4	50.6
	3.0x	20.2	206.3	148.8	74.4
	4.5x	10.1	31.8	172.4	140.2
	6.0x	15.6	21.8	56.9	185.8

PGA		cell extract (mg/mL)			
		20	40	60	80
NTP	1.5x	69.1	71.3	63.3	27.6
	3.0x	50.4	185.6	156.4	44.1
	4.5x	15.7	141.6	219.1	164.9
	6.0x	14.1	36.0	113.5	192.0

PEP		cell extract (mg/mL)			
		20	40	60	80
NTP	1.5x	51.4	58.1	28.0	44.8
	3.0x	42.6	86.8	94.1	11.8
	4.5x	6.8	110.3	141.9	95.4
	6.0x	0.0	33.6	157.9	72.5

Day 3

Glucose		cell extract (mg/mL)			
		20	40	60	80
NTP	1.5x	27.3	75.3	122.8	30.4
	3.0x	0.0	42.5	157.4	20.0
	4.5x	0.0	12.3	103.0	204.2
	6.0x	0.0	0.0	32.7	80.2

PGA		cell extract (mg/mL)			
		20	40	60	80
NTP	1.5x	39.1	71.6	49.1	28.8
	3.0x	30.9	114.3	52.0	31.9
	4.5x	16.8	66.0	146.2	109.0
	6.0x	14.2	69.1	78.1	165.9

PEP		cell extract (mg/mL)			
		20	40	60	80
NTP	1.5x	23.2	38.8	34.4	19.0
	3.0x	35.4	48.5	41.7	26.6
	4.5x	13.5	74.8	84.6	35.2
	6.0x	7.4	15.5	53.0	59.8

sfGFP (µg/mL)
0
220

Supplementary Figure S2. Energy source dependence of high concentration cell extract on CFPS activity. Raw data of Figure 4 are shown. The values indicate the levels of sfGFP synthesis (µg/mL).

Day 1

sfGFP (µg/mL)		cell extract (mg/mL) 20	40	60	80
ATP (mM)	0	69.69	99.04	50.91	32.76
	5	48.81	117.82	98.22	69.72
	10	30.32	75.81	128.49	109.64
	15	22.63	38.92	120.86	122.46
	20	22.56	32.66	81.66	120.79
ADP (mM)	0	69.69	99.04	50.91	32.76
	5	80.56	113.70	100.50	69.80
	10	44.24	111.45	145.83	98.18
	15	27.06	59.64	149.64	129.35
	20	24.58	40.90	121.15	154.98
dATP (mM)	0	69.69	99.04	50.91	32.76
	5	38.92	87.99	111.58	85.24
	10	16.48	44.95	106.27	88.13
	15	17.17	27.25	66.88	85.63
	20	20.33	33.17	41.73	47.36
Adenosine (mM)	0	69.69	99.04	50.91	32.76
	5	77.57	109.32	82.74	58.06
	10	91.00	126.44	97.82	75.36
	15	87.80	136.39	109.46	71.19
	20	85.73	147.95	108.04	88.42

Day 2

sfGFP (µg/mL)		cell extract (mg/mL) 20	40	60	80
ATP (mM)	0	28.51	38.93	28.97	12.51
	5	12.48	77.09	59.38	37.96
	10	6.96	35.18	80.59	51.69
	15	2.60	17.37	49.82	55.73
	20	6.65	8.91	24.69	39.59
ADP (mM)	0	28.51	38.93	28.97	12.51
	5	25.01	74.80	62.35	30.25
	10	2.53	87.01	90.19	45.10
	15	3.27	34.01	77.98	71.09
	20	3.53	17.16	53.46	63.51
dATP (mM)	0	28.51	38.93	28.97	12.51
	5	14.53	54.47	54.81	27.94
	10	6.22	21.29	44.29	39.94
	15	6.25	9.05	32.52	34.84
	20	15.14	8.64	13.14	14.95
Adenosine (mM)	0	28.51	38.93	28.97	12.51
	5	58.19	67.07	55.08	18.17
	10	44.22	83.05	70.23	27.77
	15	39.47	94.38	77.96	26.54
	20	36.93	93.09	75.24	36.79

Day 3

sfGFP (µg/mL)		cell extract (mg/mL) 20	40	60	80
ATP (mM)	0	52.59	84.47	73.48	19.43
	5	63.66	107.08	111.38	64.32
	10	31.72	52.42	59.88	82.10
	15	1.83	13.48	27.23	58.22
	20	-0.11	13.52	22.42	63.79
ADP (mM)	0	52.59	84.47	73.48	19.43
	5	81.63	122.68	105.25	48.53
	10	63.44	85.69	110.24	93.87
	15	61.00	76.69	114.18	92.74
	20	12.62	23.97	54.19	96.95
dATP (mM)	0	52.59	84.47	73.48	19.43
	5	20.01	57.92	74.66	55.12
	10	3.77	16.67	14.72	62.61
	15	2.51	6.53	12.05	45.79
	20	3.72	5.95	14.35	28.64
Adenosine (mM)	0	52.59	84.47	73.48	19.43
	5	88.37	128.70	108.10	24.10
	10	107.33	145.59	125.45	44.38
	15	138.52	150.20	135.95	50.07
	20	101.89	117.41	112.10	44.88

Supplementary Figure S3. Effect of ATP and its derivative of high concentration cell extract on CFPS activity. Raw data of Figure 5 are shown. Setting of the heat map is the same as in Figure 5. The values indicate the levels of sfGFP synthesis (µg/mL).

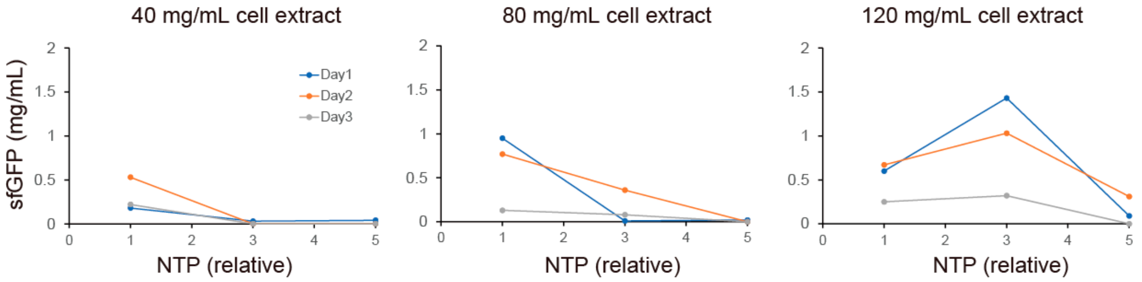

Supplementary Figure S4. NTP requirement of high concentration cell extract for cell-free protein synthesis. Raw data of dialysis-mode results in Figure 6 are shown.
